# Item response theory-based validation of a short form of the Eating Behavior Scale for Japanese adults

**DOI:** 10.1097/MD.0000000000008334

**Published:** 2017-10-20

**Authors:** Jun Tayama, Sayaka Ogawa, Atsushi Takeoka, Masakazu Kobayashi, Susumu Shirabe

**Affiliations:** a Graduate School of Education; b Center for Health and Community Medicine, Nagasaki University; c Unit of Preventive Medicine, Nagasaki University Graduate School of Biomedical Sciences, Nagasaki, Japan.

**Keywords:** body mass index, Eating Behavior Scale, item response theory, obesity, waist circumference

## Abstract

Obesity has become a serious social problem in industrialized countries in recent years. Clinically, although the evaluation of dietary behavior abnormalities is as important as any method of risk assessment for obesity, almost all the existing scales with many items may have numerous practical clinical difficulties. In this study, we aimed to prepare a short questionnaire to assess the dietary behavior abnormalities related to obesity. A total of 1032 individuals aged 20 to 59 years participated in the present study. Using item response theory (IRT), we selected the items for a short version from among 30 items of Sakata Eating Behavior Scale (EBS), which is widely used in Japan. As a result of the IRT-based analysis on the original 30-item version, 7 items were adopted as the short version. The correlation between the total score of the original EBS and the EBS short form was extremely high (*r* = 0.93, *P* = .001). In examining the criterion validity, for all participants (n = 1032), male (n = 516), and female (n = 516), the correlation coefficients between the total score of the EBS short form and body mass index (BMI) were *r* = 0.26, *r* = 0.28, and *r* = 0.28, respectively. The results of the receiver operating characteristic analysis was performed with obesity BMI > 25 kg/m^2^ as a dependent variable, the value of the area under the curve in the ROC was significantly higher in the 7-item version than in the total score of the original items (*P* = .0005). In conclusion, the 7-item EBS short form was created. Furthermore, it was found that the EBS short form is a reliable and valid measure that can be used as an indicator of obesity in both clinical and research settings.

## Abstract 2

Supplemental Digital Content is available in the text

## 1 Introduction

Obesity has become a serious social problem in industrialized countries in recent years.^[1]^ It is a major trigger of lifestyle-related diseases, such as diabetes, dyslipidemia, and hypertension, and has been found to lead to heart disease and stroke.^[2,3]^ The Japan Society of the Study of Obesity defined a body mass index (BMI) of ≥25 kg/m^2^ as a criterion for obesity, which is defined as excessive accumulation of adipose tissue.^[4]^ Increased BMI is a risk factor for arteriosclerotic diseases such as coronary heart or vascular brain disease.^[2,3]^

Clinically, widely used assessment methods related to obesity include physical^[2,3,5]^ and nutritional methods^[6–8]^ and exercise.^[6,9]^ Various biochemical tests are used in body assessment, along with macro indicators such as body weight and BMI. In the obese state, lipid markers, hepatic function markers, and hemodynamics such as blood pressure, blood sugar, and so on show abnormal values.^[2,3,5]^ Biological factors include obesity genes and intestinal bacteria.^[10]^ The main method of nutritional assessment is to calculate calorie intake from a meal menu, as well as the amount of alcohol consumption.^[6–8]^ Questionnaires^[9]^ and equipment^[11]^ are mainly used to assess frequency and types of exercise among obese people.

It is clear that abnormalities within the human mind and behavior are involved in the formation of obesity.^[12–14]^ Therefore, in order to prevent and reduce obesity by promoting the transformation of dietary behavior, assessment that includes aspects of food-related psychology and behavior is indispensable. The target of behavioral and cognitive behavioral therapy for obese people is their eating behavior, and the outcome is BMI, body weight, etc.^[15–17]^ Assessment of eating behaviors is considered extremely important to understand how the mediating variable of eating behavior changes with nonmedicinal therapy and how it affects outcomes.

There are several reliable measures for assessing the dietary behavior abnormalities associated with obesity.^[18–20]^ The Dutch Eating Behavior Questionnaire (DEBQ) is famous internationally as a widely used scale.^[18]^ The DEBQ is a measure used to assess restrained, emotional, and external eating behaviors. Sakata Eating Behavior Scale (EBS)^[19,21]^ was created and is widely used in Japan. With the EBS, it is possible to assess 7 areas concerning cognition of constitution, motivation for eating, substitute eating and drinking, feelings of satiety, eating style, meal contents, and eating rhythm abnormalities. The reliability and validity of both scales are above the recommended level. However, the DEBQ has 33 items, and the EBS has 30 items; scales with these many items may have numerous practical clinical difficulties.

In terms of the user who answers the questionnaire, when there are many items, the time and mental costs are both high, so this is a matter that needs consideration. Research into how to improve the problem of the number of items on dietary behavior scales has been increasing in recent years, and the popularization of technology has led to the creation of short forms of such questionnaires. Specifically, abbreviated versions based on item response theory (IRT) have become widely available in recent years. In relation to the rating scale method used for the assessment of eating behaviors, in the mental health domain, the SF-36,^[22,23]^ which measures health-related quality of life through 36 items was modified based on IRT to the SF-12,^[24]^ consisting of 12 items. In addition, questionnaires such as the K6 or K10 are currently widely used internationally in the assessment of psychological distress, and these both reduce the burden on the user with their reduced numbers of items.^[25]^ However, with regard to questionnaires measuring the dietary behaviors related to obesity, no scale has yet been developed to include a reduced number of items.

Therefore, in this study, we aimed to prepare a short form questionnaire to assess dietary behavior abnormalities related to obesity. Specifically, we developed a shortened version of the EBS created by Sakata et al.^[19]^ The hypotheses of this study were as follows:

1) The total scores of the EBS short form and the original EBS are strongly correlated.
2) The total EBS short form score correlates with BMI and waist circumference.
3) In the receiver operating characteristic (ROC) analysis, with the EBS short form total score and the original EBS as the independent variables and obesity state as the dependent variable, the calculated area under the curve is significantly larger for the EBS short form compared to the original EBS.

## 2 Subjects

A total of 1032 individuals aged 20 to 59 years took part in the present study (M = 40, SD = 11). They consisted of 516 women and 516 men. There was no significant difference in age between the genders (*t*(1030) = 1.96, *P* = .41, *d* = 0.00). No specific inclusion or exclusion criteria were used. The study protocol was approved by the Ethics Committee of Nagasaki University (no. 17033113).

## 3 Materials and methods

### 3.1 Demographic variables

All participants were asked to respond to the questions about their age, gender, height, weight, and waist circumference. The data on participants’ height, weight, and waist circumference were to be obtained based on self-reported medical examination results within the past year.

### 3.2 Provisional version of the EBS short form

Thirty items of Sakata EBS (“Eating Behavior Questionnaire”, in its original form)^[19,21,26]^ are widely used in Japan. These 30 items are classified into 7 areas concerning cognition of constitution (3 items), motivation for eating (3 items), substitute eating and drinking (6 items), feeling of satiety (5 items), eating style (3 items), meal contents (5 items), and eating rhythm abnormalities (5 items). The total score for each area of the EBS original version is different between obese and healthy subjects.^[19,21]^ Responses were chosen from 4 options: “strongly disagree”, “somewhat disagree”, “somewhat agree”, and “strongly agree”. Higher scores indicated abnormal eating behaviors that reinforce and worsen obesity. The provisional version of EBS short form adopted 29 items (third item pool), excluding “Cannot chew well”, which is an item in the eating style area.

### 3.3 Survey procedure

We conducted online surveys in January 2017. Participants were recruited from an online panel database provided by a Japanese Internet research company, Macromill, Inc. This database had over 370,000 individual users at the time of this survey between the ages of 20 and 59 years who were registered as research volunteers. An equal number of participants, with equal gender distribution, were assigned to each age group ranging in age from the 20s to the 50s.

This study conformed to the ethical guidelines mentioned in the Helsinki Policy Statements, which are comparable to guidelines followed by institutional review boards at U.S. universities. First, participants were instructed as to the research aim and the intended use of the survey data. They were guaranteed anonymity, should they decide to take part. Individuals who agreed to the stated procedures and conditions were able to participate in the present study. After they provided their consent, the participants filled out demographic questions and completed the provisional version of the EBS short form on the Internet. After completing the questionnaires, each of them received approximately 50 cents U.S. as pay for their participation through the Macromill, Inc., system. Since individual data were acquired through an Internet research company, data from this study are not appropriate for public deposition. With regard to data availability, all relevant data are included within the paper.

### 3.4 Analysis procedure

#### 3.4.1 Examination of content validity

For each area, the primary item pool is divided into cognition of constitution (3 items), motivation for eating (3 items), substitute eating and drinking (6 items), feeling of satiety (5 items), eating style (3 items), meal contents (5 items), and eating rhythm abnormalities (5 items). Next, for the primary item pool, 1 physician and 1 clinical psychologist determined the items’ adequacy for measuring the extent of adult eating behavior abnormality, and they created 29 items for the second item pool. Then, as to the secondary item pool, 1 physician and 1 clinical psychologist determined whether the items for measuring the extent of adult eating behavior abnormality were adequate, with ratings from “1: Inadequate” to “4: It is reasonable”. As a result, 29 items with a median value of 3 or more were combined to form the third item pool of the provisional version of the EBS short form, as shown in Table 1.

**Table 1:**
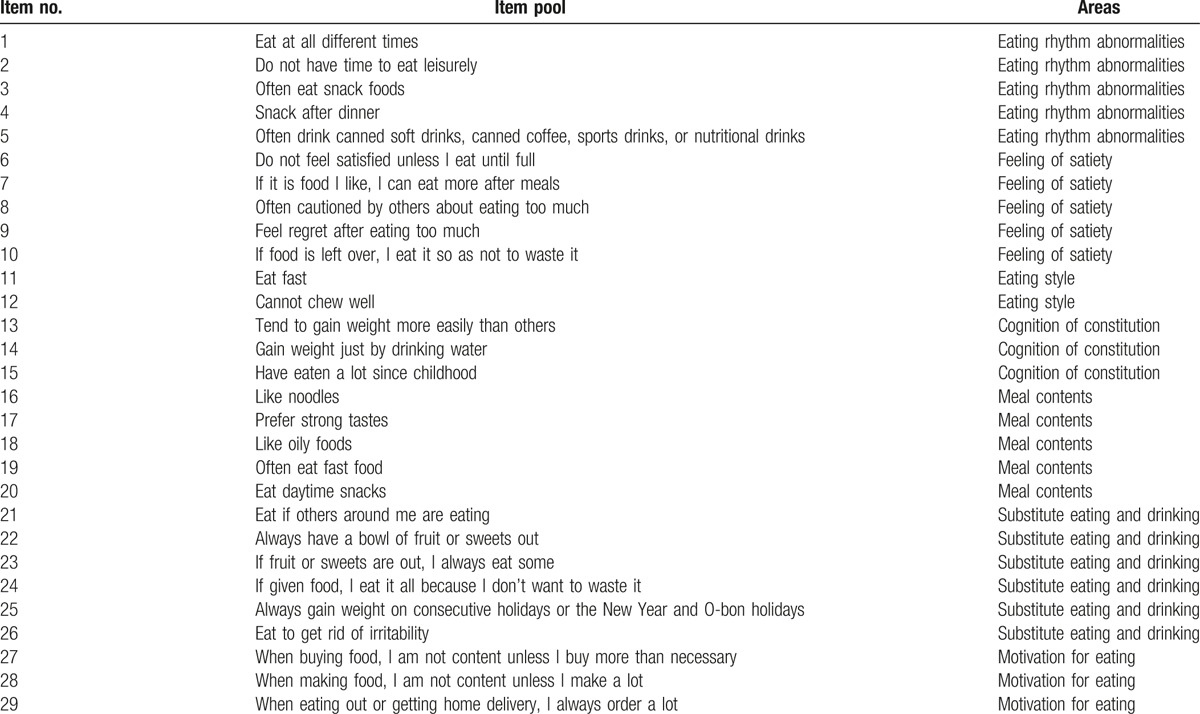
Provisional version of the EBS short form (29 items).

#### 3.4.2 Confirmation of 1-dimensionality

To understand the responses of 1032 subjects to the provisional version of the EBS short form, factor analysis (principal factor method) was performed for each area, and the attenuation situation of eigenvalues for the correlation matrix was examined.

#### 3.4.3 Selection of items

In the factor analysis results, the factor loading of the item was conditioned to be 0.40 or more. Furthermore, based on the IRT, the discrimination ability “a” of each item was calculated. Items were selected based on *a* > 0.75^[27,28]^ and based on the discrimination Top 1. Items selected by the above procedure were set as items of the EBS short form.

#### 3.4.4 Examination of criterion validity

Correlation analysis between the total score of the original EBS and the EBS short form was performed. We calculated the correlation coefficient between the total score of the EBS short form and BMI and waist circumference among both sexes. Furthermore, ROC analysis was performed with obesity BMI > 25 kg/m^2^ as a dependent variable.

#### 3.4.5 Statistical analysis

For the statistical analysis, SPSS (ver. 21.0) and R (ver. 3.3.2) were used. In this study, since there were 4 response methods in the IRT, a graded response model, GRM,^[29]^ was used. We used the “ltm (Latent Trait Models)” package in R when running IRT.

## 4 Results

One item was excluded from the 1st and 2nd item pools. The excluded item was “Cannot chew well”, an item that was presumed to be more applicable to the elderly. This study's subjects were 20 to 59 years old, and since no elderly people were included in the participant pool, this item was deleted at the time of item selection.

As shown in Table 1, for the provisional version of the EBS short form of 29 items, it was judged to be an appropriate question by 2 experts for item evaluation. They quantitatively examined all the items and sorted them on the basis of the median. Through such procedures, 29 items remained. Therefore, we concluded that the content validity is guaranteed.

Table 2 shows the results of checking the 1-dimensional property by factor analysis (principal factor method) for the responses obtained from the subjects. As a result of checking the attenuation situation of the eigenvalues of the correlation matrix, it became clear that 1 dimensionality is maintained in all areas. As a prerequisite for conducting analysis based on IRT, the 1 dimensionality of each area needs to be confirmed. It turned out that this condition was satisfied by analyzing the attenuation situation of the eigenvalues of the correlation matrix. Furthermore, since the factor loading in all the items in each area was 0.6 or more, there were no items corresponding to the exclusion criteria in the factor loading.

**Table 2:**
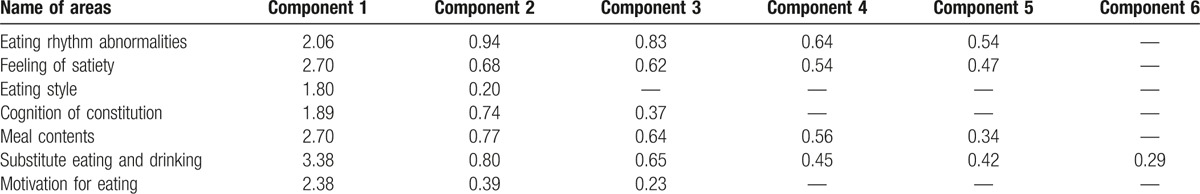
Eigenvalues of the correlation coefficient matrix.

For the 29 items that were not excluded, the values of the discriminatory “*a*” and difficulty “*b*” parameter for each item were calculated for each area based on IRT (Table 3). Items 5 and 15 did not satisfy the condition of *a* > 0.75. Ultimately, 1 item with the highest discrimination power was selected from each area, and all 7 items were adopted for the EBS short form (Appendix).

**Table 3:**
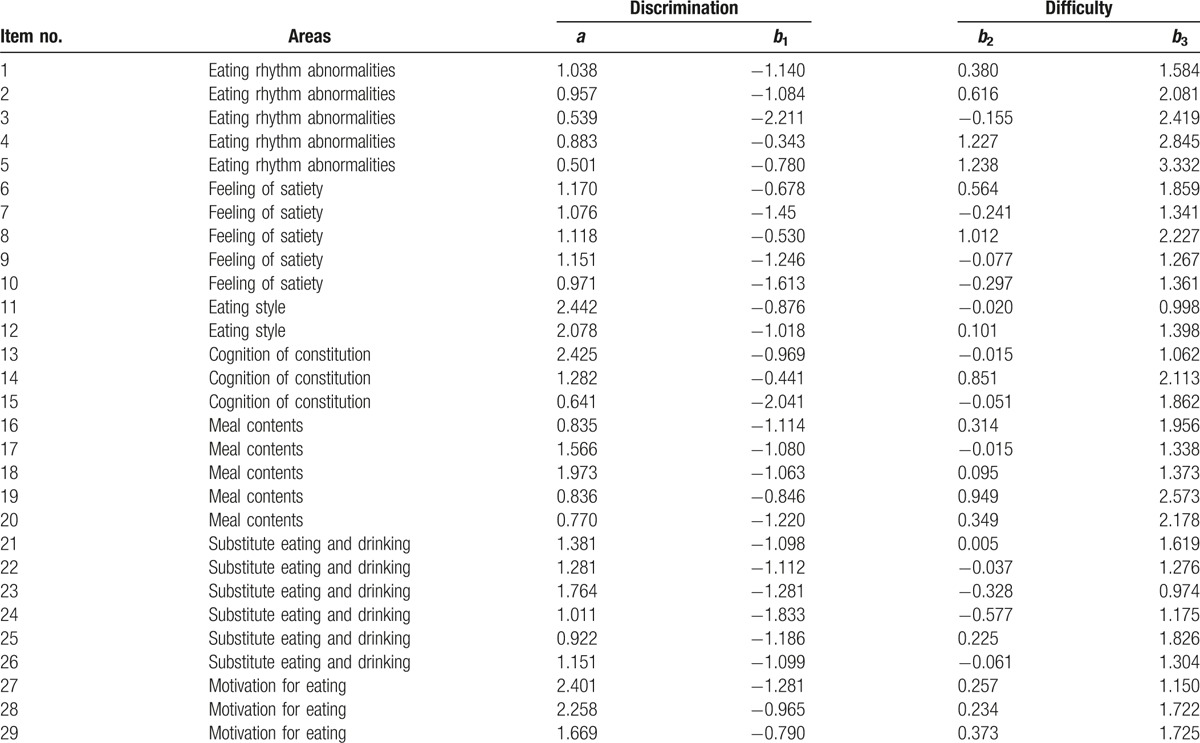
Discrimination and difficulty parameters for each item.

**Figure 1:**
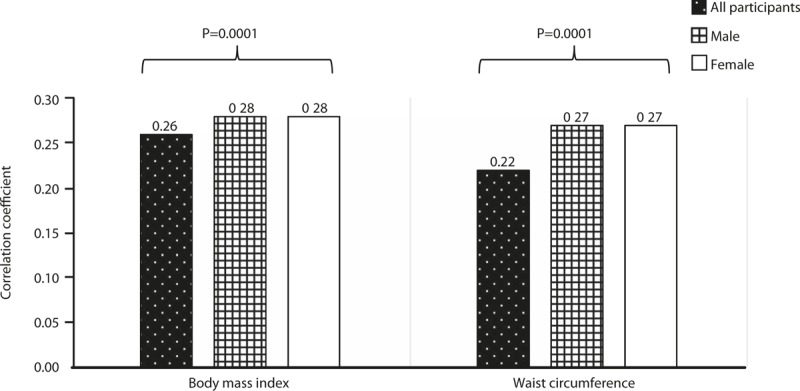
Correlation coefficients between the total score of the EBS short form and BMI and waist circumference.

The correlation between the total score of the original EBS and the EBS short form was extremely high (*r* = 0.93, *P* = .001). For the examination of the criterion validity, for all participants (n = 1032), male participants (n = 516), and female participants (n = 516), the correlation coefficients between the total score of the EBS short form and BMI were *r* = 0.26, *r* = 0.28, and *r* = 0.28, respectively, while the correlation coefficients between the total score of the EBS short form and the waist circumference were *r* = 0.22, *r* = 0.27, and *r* = 0.27, respectively (Fig. 1). From these results, since a positive correlation was found between the total score of the EBS short form and obesity-related indices, criterion validity was confirmed.

**Figure 2:**
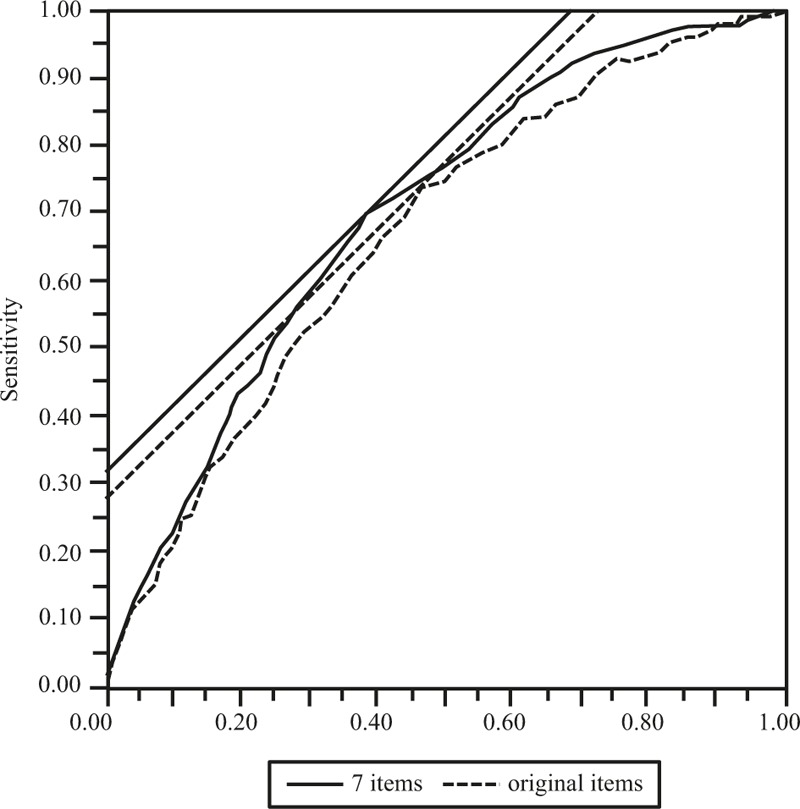
Receiver operating characteristic (ROC) analysis with the total score of the EBS short form and the original EBS as the independent variables and obesity state as the dependent variable.

The following values were derived by the survival analysis, using the total score of the 29 items as an explanatory variable: sensitivity = 0.74, 1-specificity (false-positive rate) = 0.28, and the area under the curve = 0.67 (95% confidence interval (CI), 0.63–0.71) (Fig. 2). In the survival analysis using the total score of the 7 items as an explanatory variable, the following values were derived: sensitivity = 0.70, 1-specificity (false-positive rate) = 0.32, and area under the curve = 0.70 (95% CI, 0.66–0.74). The value of the area under the curve in the ROC was significantly higher in the 7-item version than in the total score of the original items (*P* = .0005).

## 5 Discussion

The results of this study showed that the total score of the EBS short form and that of the original EBS were strongly correlated. Additionally, the total score of the EBS short form was correlated with BMI and waist circumference. Furthermore, in the ROC analysis, with the total score of the EBS short form and the original EBS as the independent variables and obesity state as the dependent variable, the calculated area under the curve was slightly or significantly larger in the EBS short form compared with that of the original EBS. Therefore, this study supported the 3 hypotheses.

The reason why the total score of the EBS short form and that of the original EBS were strongly correlated is that the degree of total eating behavior abnormality measured by the EBS short form has nearly the same meaning for the degree of total dietary behavior abnormality as that measured by the original version of the EBS. Moreover, the quality of the total dietary behavior abnormalities measured by the EBS short form would be approximately equivalent to that of the original. In a previous study that examined whether the concept of each area of the EBS original version would be a risk factor for obesity, it was shown that all areas are, in fact, indicative of obesity risk.^[30]^

Since the total score of the EBS short form was correlated with BMI and waist circumference, we could mention that the criterion validity of the EBS short form was good. The correlation coefficients in the total score of the EBS in both BMI and waist circumference were lower than in the correlation coefficient for each gender. In some epidemiological studies, it has been reported that both BMI^[31,32]^ and waist circumference^[33,34]^ values of men are higher than for women. In other words, the reason for the low correlation coefficient of all participants is mainly because of the binomial distribution of data for both BMI and waist circumference. Regarding BMI and waist circumference being selected for the examination of criterion validity regarding body indicators, BMI is in particular an indicator of the gold standard of obesity determination. The positive correlation between the total score of the EBS short form and BMI in this study showed that the criterion validity of the EBS short form is above an acceptable level.

For the ROC analysis with the total form of the EBS short form and the original EBS as the independent variables and obesity state as the dependent variable, the calculated area under the curve was significantly larger in the EBS short form compared with the original EBS. This guarantees the high criterion validity of the EBS short form, just as was the case with the correlation found between the total score of the EBS short form and BMI and waist circumference. In other words, the results of the ROC analysis showed that the EBS short form is also an acceptable measure of eating behaviors associated with obesity. In addition, it was suggested that the EBS short form is useful for predicting obesity.

The advantage of this research is that we created scales that can be utilized by men and women of various age groups. We conducted a survey with homogeneous samples at a ratio of 5: 5 for males and females, respectively, in each decade of the age span of 20 to 50 years. Therefore, the EBS short form is considered to be usable for men and women in their 20s to 50s. Moreover, by using the EBS short form, their common eating behavior abnormalities can be assessed. Assessment of dietary behavior abnormalities is essential for the prevention of and decrease in obesity. In doing this, the staff members who support patients will be able to assess eating behavior abnormalities by utilizing the EBS short form. There are 2 major methods of evaluating eating behavior abnormalities. The first is to utilize the EBS short form in screenings such as in diagnosis or medical examinations. The second is to make eating behavior abnormalities evaluation useful for individual support. In 1 intervention study, the total score of the original version of the EBS was shown to decrease as BMI decreased.^[35]^ For those with dietary behavior abnormalities associated with obesity risk, self-understanding of such abnormalities may improve them, thereby enhancing the individuals’ physical health.

This study has a couple of limitations. The first is that this research was a web-based survey, and we have not obtained biochemical test data such as lipids, leptin, and blood sugar, or body fat data, etc. Dietary behavior abnormalities are known to cause abnormalities in various bio markers.^[12,14]^ Eating behavior abnormalities are expected to mediate some bio markers, resulting in obesity. In the future, by using the EBS short form and biochemical test data, further verification of the reliability of the EBS short form will be necessary. Second, the EBS short form is not intended for those under the age of 20 or over the age of 60. The prevalence of obesity in different generations is different.^[36]^ It is known that dietary behavior abnormalities including irregular meals are prevalent even in middle-aged and elderly people.^[37]^ In the future, it will be necessary to consider the eating behaviors of these populations.

Clinically, various factors are involved in obesity. Among these, eating behavior abnormality is a major risk of obesity.^[26,30,38]^ Evaluations of dietary behaviors are indispensable for research and practice using indicators such as an increase or a decrease in weight. Indeed, the original version of the EBS has also been used as an outcome for cohort studies^[30]^; the short form of this study may be useful for outcomes of observation or intervention studies. In addition, this scale may be useful as an index for measuring eating behaviors that are targets and parameters in intervention studies with body weight or BMI as outcomes.

In conclusion, the 7-item EBS short form was created. Furthermore, it became clear that the EBS short form is a reliable and valid measure that can be tolerated as an indicator of obesity in both clinical and research settings.

## Supplementary Material

Supplemental Digital Content
