# Supplementary material for: Item response theory-based validation of a short form of the Eating Behavior Scale for Japanese adults
Source: Medicine (Baltimore). 2017 Oct 20;96(42):e8334. doi: 10.1097/MD.0000000000008334 (PMC5662414; doi:10.1097/MD.0000000000008334)
Supplement: Supplemental Digital Content [file medi-96-e8334-s001.docx]

APPENDIX

For each item, please answer from 1 (strongly disagree) to 4 (strongly agree).

4. strongly agree, 3. somewhat agree, 2. somewhat disagree, 1. strongly disagree

**Eating rhythm abnormalities**

*1. Eat at all different times.

2. Do not have time to eat leisurely.

3. Often eat snack foods.

4. Snack after dinner.

5. Often drink canned soft drinks, canned coffee, sports drinks, or nutritional drinks.

**Feeling of satiety**

*1. Do not feel satisfied unless I eat until full.

2. If it is food I like, I can eat more after meals.

3. Often cautioned by others about eating too much.

4. Feel regret after eating too much.

5. If food is left over, I eat it so as not to waste it.

**Eating style**

*1. Eat fast.

2. Cannot chew well.

**Cognition of constitution**

*1. Tend to gain weight more easily than others.

2. Gain weight just by drinking water.

3. Have eaten a lot since childhood.

**Meal contents**

1. Like noodles.

2. Prefer strong tastes.

*3. Like oily foods.

4. Often eat fast food.

5. Eat daytime snacks.

**Substitute eating and drinking**

*1. Eat if others around me are eating.

2. Always have a bowl of fruit or sweets out.

3. If fruit or sweets are out, I always eat some.

4. If given food, I eat it all because I don’t want to waste it.

5. Always gain weight on consecutive holidays or the New Year and O-bon holidays.

6. Eat to get rid of irritability.

**Motivation for eating**

*1. When buying food, I am not content unless I buy more than necessary.

2. When making food, I am not content unless I make a lot.

3. When eating out or getting home delivery, I always order a lot.

*Adopted items as a shortened version.
